# Cerium Dioxide-Based Scavenging of Environmentally Persistent Free Radicals in Fine Particulate Matter: A Prospective Review

**DOI:** 10.3390/ijms27209232

**Published:** 2026-10-17

**Authors:** Qingyang Liu, Zheng Yang, Yanju Liu

**Affiliations:** 1 College of Ecology and the Environment, Nanjing Forestry University, Nanjing 210037, China; 2 Beijing Milu Ecological Research Center, Beijing 100076, China; 3 Institute of Analysis and Testing, Beijing Center for Physical and Chemical Analysis, Beijing Academy of Science and Technology, Beijing 100089, China

**Keywords:** CeO_2_, environmentally persistent free radicals (EPFRs), PM_2.5_, air filtration, reactive oxygen species, antioxidant nanomaterial

## Abstract

Environmental persistent free radicals (EPFRs) are a class of long-lived, redox-active species that persist on fine particulate matter (PM_2.5_) surfaces for days to months. Upon inhalation, EPFRs catalyze the generation of hydroxyl radicals through Fenton-like cycling, driving oxidative stress and inflammation at the site of deposition. Current air filtration technology focuses almost exclusively on physical interception of particulate matter, leaving the surface-bound radical activity of captured PM entirely unaddressed. This review advances a testable hypothesis rather than reporting a validated outcome: that cerium dioxide (CeO_2_)-based materials, whose Ce^3+^/Ce^4+^ redox cycling is well-established as a broad-spectrum antioxidant mechanism in biomedical contexts, can be functionally migrated to air filtration to chemically quench EPFRs on captured PM_2.5_. We first summarize the formation and health impacts of EPFRs, then introduce the mechanistic basis of CeO_2_ radical scavenging, emphasizing its self-regenerating valence cycling. We then examine the existing but functionally distinct use of CeO_2_ in air pollution control, including its role as an oxygen storage component in three-way catalysis and as an additive in filter media, and argue that these high-temperature oxidation applications do not constitute a functional precedent for ambient-temperature radical scavenging. The central gap is that no study has systematically tested whether CeO_2_ can chemically quench EPFRs on PM_2.5_ surfaces under filtration conditions. We articulate the key experimental questions that must be resolved—direct EPFR quenching versus ROS interception, the effects of humidity, airflow, and contact time, and the long-term fate of CeO_2_ under realistic filtration conditions—and argue that the demonstrated gaseous ROS scavenging of CeO_2_ in cigarette smoke, together with its self-recovery capability, provides sufficient mechanistic justification to pursue this direction experimentally.

## 1. Introduction

Ambient particulate matter with aerodynamic diameter ≤ 2.5 μm (PM_2.5_) remains one of the most significant environmental risk factors for global morbidity and mortality [1]. While the mass concentration of PM_2.5_ is the primary regulatory metric, a growing body of evidence indicates that the chemical composition and surface reactivity of particles, rather than mass alone, drive many of the observed health effects [2,3].

Among the surface-associated species that contribute to PM_2.5_ toxicity, environmentally persistent free radicals (EPFRs) are increasingly recognized as a critical class of pollutants [4]. EPFRs are stable free radicals formed on particulate matter through combustion and pyrolysis processes, generated on transition metal oxide surfaces in the presence of aromatic precursors [5]. Unlike transient atmospheric radicals (e.g., ·OH, with lifetimes of microseconds to seconds), EPFRs are stabilized on particle surfaces through interactions with transition metal oxides or within organic matrices [6]. Their half-lives can range from several weeks to, in some cases, several years [7]. EPFRs may be carbon-centered, oxygen-centered, or mixed, identified by g-values exhibited in electron paramagnetic resonance (EPR) analysis [8]. These radicals persist long enough to be transported, inhaled, and deposited in the respiratory tract [9]. Beyond EPR, chemiluminescence and bioluminescence techniques provide complementary approaches for detecting radical activity and oxidative stress in environmental samples. Chemiluminescence probes can report the generation of reactive oxygen species through light emission resulting from radical–probe reactions, and they are sufficiently sensitive to detect low-level ROS production in complex matrices [2]. Bioluminescence-based methods, particularly those using luminescent bacteria, offer an integrative measure of toxicity and oxidative stress: the bacterial luminescence response reflects the overall biological impact of radical-generating species rather than the concentration of a single radical type [4]. These methods are not replacements for EPR, which remains the definitive technique for identifying and quantifying EPFRs by g-value and spin concentration [2,8]. Rather, they are complementary: EPR provides radical-specific information, whereas chemiluminescence and bioluminescence provide functional information about ROS generation and biological response. For the present review, these techniques are relevant in two ways. First, they can be used in the experimental framework proposed in Section 7 as rapid screening tools to assess whether CeO_2_ treatment reduces the net ROS-generating capacity of EPFR-bearing PM_2.5_. Second, they can help bridge the gap between radical quantification and biological effect, because a reduction in EPR signal does not necessarily translate into a proportional reduction in oxidative stress if the remaining radicals or co-existing species still drive ROS production. Including chemiluminescence and bioluminescence assays alongside EPR and acellular oxidative-potential assays would therefore provide a more comprehensive evaluation of CeO_2_ performance under realistic filtration conditions [2,4].

Exposure to EPFRs occurs primarily via inhalation of PM_2.5_ deriving from combustion, traffic, industrial activities, and both indoor and outdoor burning [10]. Other environmental sources include radon, electronic cigarettes, and tobacco smoke [11]. EPFR concentrations in these sources vary widely, ranging from 10^17^ spins/g to 10^23^ spins/g, depending on the type, location of the pollutant source, and season [12]. Notably, a study by Lee et al. (2025) identified several household practices correlated with EPFR concentrations, including construction type, exposure to traffic-related air pollution, indoor combustion activities, and ventilation, demonstrating the dependence of indoor air quality upon household practices [13]. It should be mentioned that environmental pollution contains not merely persistent free radicals, but also efficient scavengers of free radicals. Such scavengers may be of technogenic origin, as documented for certain photochemically active industrial pollutants [14], and may also be derived from cigarette smoke [15]. The co-occurrence of EPFRs and free-radical scavengers in the same environmental matrix represents a noticeable challenge for ecotoxicology [5,9]. The net oxidative potential of a particle or gas mixture is not simply the sum of its radical content; it depends on the balance between radical-generating species and radical-quenching species [3,9,11]. If efficient scavengers are present, they may partially mask the biological effects of EPFRs in acellular or cellular assays, leading to underestimation of EPFR toxicity [3,4,5]. Conversely, if the scavengers are consumed or degraded more rapidly than EPFRs, the radical activity may become more pronounced over time [7,8,12]. This coexistence complicates both the interpretation of oxidative-potential measurements and the design of mitigation strategies, because a material intended to quench EPFRs must function in a chemical environment that already contains competing radical scavengers [3,5,9]. It also reinforces the need for source-specific characterization: the ratio of EPFRs to radical scavengers may differ substantially between combustion sources, indoor environments, and cigarette smoke [3,10,11,15]. For the hypothesis advanced in this review, this issue has two implications. First, the baseline radical-scavenging capacity of the PM_2.5_ matrix itself must be characterized before the incremental effect of CeO_2_ can be quantified. Second, the performance of CeO_2_ should be evaluated not only against EPFR concentration but also against the net oxidative potential of the treated particles, because the latter integrates both radical generation and radical quenching [3,9]. Recognizing the presence of endogenous and technogenic radical scavengers therefore strengthens the experimental framework proposed in Section 7 and helps avoid overestimating the apparent effect of CeO_2_ in complex environmental samples.

The mechanism linking EPFRs to adverse health outcomes centers on their capacity to catalyze the generation of reactive oxygen species (ROS), particularly hydroxyl radicals (·OH) [14]. EPFRs adsorbed on PM_2.5_ undergo redox cycling in the presence of molecular oxygen and biological reducing agents, producing superoxide O_2_^−^, hydrogen peroxide H_2_O_2_, and ultimately ·OH through Fenton-like chemistry [15]. The radicals undergo redox cycling within aqueous solutions and in biological tissues/fluids, triggering production of ROS comprised primarily of hydroxyl, superoxide, and peroxyl radicals [16]. This sustained ·OH production at the particle surface is hypothesized to drive oxidative damage to lipids, proteins, and DNA in the lung epithelium and beyond [17]. Toxicological data in animals suggest that EPFR inhalation contributes to cardiovascular, respiratory, and metabolic diseases [18]. However, it is noted that EPFRs are not currently surveyed by any regulatory monitoring system [19]. This regulatory gap, combined with the documented persistence and toxicity of EPFRs, underscores the urgent need for mitigation strategies [20].

Current air filtration technologies, from fibrous mats to high-efficiency particulate air (HEPA) filters, are engineered to maximize particle capture through mechanisms including interception, inertial impaction, and electrostatic attraction [21]. These approaches are highly effective at removing PM_2.5_ from the airstream. However, they operate on a fundamentally passive logic: once a particle is captured, it remains on the filter surface, and any radical activity associated with that particle persists [22]. This functional limitation has a direct consequence: the filter becomes a concentrated reservoir of EPFR-bearing particles. While some radical decay occurs over time, the chemical quenching of EPFRs by the filter material itself is not a design objective [23]. The absence of an active detoxification function means that filtration efficiency, measured as mass removal, may not correlate with the reduction in the oxidative potential of the treated air [24]. The EPFR studies have made significant advances in understanding formation mechanisms, analytical detection methodologies, environmental distribution patterns, and toxicological impacts [25]. However, none of these reviews addresses the removal or chemical quenching of EPFRs by filtration materials. This review asks whether advanced functional materials can bridge this gap. Specifically, we examine whether CeO_2_ can be integrated into filtration media to confer an active detoxification function on captured PM_2.5_ [26].

The selection of CeO_2_ as the focal material for this review is not arbitrary. It rests on three distinctive features. First, CeO_2_ possesses a self-regenerating Ce^3+^/Ce^4+^ redox cycle that enables sustained radical scavenging without the stoichiometric consumption that limits conventional antioxidants [27]. Cerium is the most abundant rare earth metal and exists in +3 and +4 oxidation states; at the nanoscale, cerium oxide contains a mixture of cerium in +3 and +4 oxidation states on nanoparticle surfaces, with the number of Ce^3+^ sites increasing as nanoparticle diameter decreases and oxygen atoms are lost [28]. Under oxidative conditions, Ce^3+^ is oxidized to Ce^4+^, but the material can subsequently re-reduce to Ce^3+^ through interaction with the surrounding environment [29]. This cycling has been directly observed: after treatment with H_2_O_2_, CeO_2_ undergoes a visible color change from pale yellow to orange, indicating oxidation of Ce^3+^ to Ce^4+^ [30]. This is followed by a spontaneous return to the original pale yellow over approximately six days, with XPS-confirmed recovery of the Ce^3+^/Ce^4+^ ratio [31]. This self-recovery distinguishes CeO_2_ from molecular antioxidants such as vitamin E, which are consumed upon reaction [32].

Second, CeO_2_ exhibits broad-spectrum activity against multiple ROS. Its enzyme-mimetic activities include superoxide dismutase (SOD)-like, catalase (CAT)-like, and direct ·OH scavenging capabilities [33]. The catalytic behavior of CeO_2_ nanoparticles illustrates that nanozyme behavior cannot be reduced to a single antioxidant label: enzyme-like activity depends significantly on nanoparticle composition, the ratio between surface oxidation states, particle size, and pH [34]. CeO_2_ nanoparticles act principally as antioxidant nanozymes, with SOD-like activity favored at higher Ce^3+^/Ce^4+^ ratios [35]. The pH-dependent switch between SOD-like and CAT-like activity reflects the tunability of its surface chemistry, and its capacity to scavenge ·OH is particularly relevant because biological systems lack a dedicated enzymatic pathway for ·OH elimination [36].

Third, CeO_2_ has already demonstrated radical scavenging activity in a relevant gas-phase context. This establishes a precedent, albeit in a different application context, for CeO_2_ functioning as a radical scavenger under airflow conditions [32]. Cigarette smoke represents an extreme scenario with high radical concentrations and transient exposure; if CeO_2_ can function under such conditions, its migration to lower-concentration, longer-duration indoor PM_2.5_ filtration scenarios is mechanistically plausible [36].

Furthermore, existing CeO_2_ applications in air pollution control, particularly as an oxygen storage component in three-way catalytic converters, operate on a fundamentally different functional logic: high-temperature (200–500 °C) oxidation of pollutants [37]. The radical scavenging function proposed here operates at ambient temperature and targets existing radicals rather than oxidizing stable molecules [38]. This functional distinction has not been previously articulated in the context of air filtration materials design [39].

This review is built upon a central hypothesis: CeO_2_-based materials, whose radical scavenging mechanisms have been extensively validated in biomedical contexts, can be functionally migrated to air filtration to chemically quench EPFRs on captured PM_2.5_, thereby extending air filtration from physical interception to active detoxification [26]. This hypothesis is motivated by three gaps in the current literature. EPFR science has thoroughly described the formation, persistence, and toxicity of EPFRs, but focuses on characterization and risk assessment rather than material-based removal [25]. CeO_2_ nanozyme science has documented catalytic mechanisms, multi-enzyme activities, and self-regeneration in biomedical applications, but remains confined to biological systems [32]. Air filtration technology has optimized particle capture, pressure drop, and mechanical stability, but its functional objectives are limited to physical interception and, in some cases, antimicrobial activity [40]. It is important to state that this is a prospective, hypothesis-driven review. It bridges three otherwise disconnected studies to articulate a testable research direction. The hypothesis remains experimentally unvalidated [41]. Our contribution is to identify the critical unknowns, specify the experimental questions that would confirm or refute the hypothesis, and provide the mechanistic justification for pursuing those experiments. This review is therefore intended to stimulate experimental investigation rather than report a validated outcome.

## 2. Literature Search Strategy

### 2.1. Search Rationale

Given the interdisciplinary nature of this review, which bridges EPFR toxicology, CeO_2_ nanozyme chemistry, and air filtration materials, the literature search was designed to capture relevant publications across three distinct domains and to identify the gaps between them. Rather than a systematic review with a single focused question, this is a narrative-critical review that synthesizes knowledge from multiple fields to articulate a new research direction. The search strategy therefore prioritized breadth across disciplines and depth within the critical intersection.

### 2.2. Databases and Search Sources

The following databases were searched: Web of Science Core Collection for comprehensive coverage of environmental science, materials science, and chemistry; PubMed/MEDLINE for EPFR toxicology and biomedical CeO_2_ applications; Scopus for cross-disciplinary coverage and citation tracking; and Google Scholar for gray literature, preprints, and citation chaining. Reference lists of key reviews and primary research articles were manually screened to identify additional relevant publications through backward citation chaining. Forward citation chaining was performed using Google Scholar and Web of Science to identify articles citing seminal papers in each domain.

Search terms were organized into three thematic clusters. The first cluster covered EPFRs and particle-bound radicals, using terms such as “environmentally persistent free radical,” “EPFR,” “particle-bound radical,” “PM_2.5_ radical,” and combinations of “free radical” with “particulate matter” or “PM_2.5_,” along with “EPR” or “electron paramagnetic resonance” and “PM_2.5_,” combined with “toxicity,” “health effect,” “ROS,” or “oxidative stress.” The second cluster covered CeO_2_ radical scavenging and nanozyme activity, using terms such as “cerium oxide,” “ceria,” “CeO_2_,” “nanoceria,” “nanozyme,” “enzyme-mimetic,” “antioxidant,” “radical scavenging,” “ROS scavenging,” “SOD,” “catalase,” “superoxide dismutase,” “hydroxyl radical,” “self-regenerating,” “redox cycling,” and “Ce^3+^/Ce^4+^”. The third cluster covered air filtration and CeO_2_ integration, using terms such as “air filtration,” “air filter,” “filtration membrane,” “nanofiber,” “electrospun,” “electrospinning,” “CeO_2_” combined with “filter,” “membrane,” or “filtration,” and “air purification” combined with “cerium” or “ceria”.

Intersection queries were performed to identify the critical gap. These included combinations of “CeO_2_,” “ceria,” or “nanoceria” with “EPFR” or “environmentally persistent free radical”; combinations of “CeO_2_” or “ceria” with “PM_2.5_” or “particulate matter” and “radical scavenging” or “detoxification”; and combinations of “air filtration” or “filter” with “radical scavenging” or “ROS scavenging” and “CeO_2_” or “ceria.”

### 2.3. Inclusion and Exclusion Criteria

Inclusion criteria covered peer-reviewed original research articles, critical reviews, and systematic reviews published in English that addressed at least one of the three thematic clusters. For EPFR literature, studies reporting concentration, half-life, health effects, or detection methods were included. For CeO_2_ literature, studies reporting radical scavenging activity, enzyme-mimetic mechanisms, or self-regeneration properties were included. For filtration literature, studies on nanofibrous membranes, air filter materials, or CeO_2_-based filtration systems were included. Exclusion criteria covered conference abstracts without full-text availability, studies not available in English, studies on unrelated nanomaterials without CeO_2_ or EPFR relevance, and purely theoretical studies without experimental validation, except for mechanistic modeling papers directly relevant to the hypothesis.

The search revealed three distinct publication patterns. The EPFR literature has grown steadily since 1998, and recent systematic reviews comprehensively cover formation mechanisms, detection methods, environmental distribution, and health effects. However, these reviews consistently identify knowledge gaps in characterization and risk assessment rather than in mitigation or removal. CeO_2_ nanozyme literature is a mature field with extensive mechanistic understanding of SOD-like, CAT-like, and ·OH scavenging activities, and self-regeneration via Ce^3+^/Ce^4+^ cycling is well documented. However, applications are overwhelmingly biomedical, including cancer therapy, neuroprotection, and anti-inflammation, with essentially no studies on air filtration. Air filtration literature has extensively optimized physical capture mechanisms and antimicrobial functionalization, and CeO_2_ has been incorporated into filtration media, but for high-temperature exhaust purification as an oxygen storage component or for antimicrobial enhancement, not for EPFR detoxification. The Boolean queries combining CeO_2_ and EPFRs returned no relevant publications. The intersection of CeO_2_ radical scavenging and PM_2.5_ EPFR removal in filtration is a genuine literature void. This void is not an oversight; it reflects the disciplinary separation between environmental toxicology, which studies EPFRs, and materials chemistry, which studies CeO_2_ nanozymes. Bridging this gap requires articulating a cross-disciplinary hypothesis, which is the central contribution of this review.

### 2.4. Review Limitations

This review is a narrative-critical synthesis rather than a systematic review with meta-analysis. The absence of quantitative pooling or risk-of-bias assessment is appropriate given the heterogeneity of the three contributing studies and the primary objective of articulating a research direction rather than synthesizing effect sizes. The search strategy prioritized sensitivity (capturing relevant publications across disciplines) over specificity, and the selection of studies for detailed discussion reflects the authors’ judgment regarding their relevance to the central hypothesis.

## 3. EPFRs and the Chemistry of Particle-Bound Radicals

### 3.1. Formation and Stabilization

EPFRs are stable free radicals formed on particulate matter (PM) surfaces through combustion and pyrolysis processes, generated on transition metal oxide surfaces in the presence of aromatic precursors [4]. EPFRs form primarily through two pathways: transition-metal-mediated stabilization and organic matrix stabilization. It is important to avoid overgeneralizing the view that EPFRs are exclusively surface-bound species generated on transition metal oxide surfaces in the presence of aromatic precursors [5,6,9,12]. This pathway is well documented and mechanistically important, but it is not the only route to EPFR formation [6,7,9]. Particles formed from incompletely combusted fuels with low metal content can also host persistent radicals, particularly when combustion is incomplete and carbonaceous residues retain aromatic domains [4,10,11,12,42]. In such particles, radicals may be stabilized within the organic matrix itself, through resonance delocalization in condensed aromatic structures, through steric protection within microporous carbonaceous domains, or through formation of covalent adducts with the matrix [9,12,42]. These matrix-stabilized radicals are not necessarily located on the particle surface; they may reside within the bulk of the particle or within internal pores that are not directly exposed to the gas phase [9,11,12,42].

This distinction has direct implications for the hypothesis advanced in this review. If a significant fraction of EPFRs is embedded within the particle bulk rather than exposed on the surface, then direct quenching by CeO_2_ would be sterically hindered or entirely prevented, because CeO_2_ cannot access radicals that are not at the particle–air interface [9,12,42]. In that case, the dominant mechanism of CeO_2_ action would necessarily be downstream interception of ROS generated by surface-accessible radicals, rather than direct quenching of the radicals themselves. This possibility is discussed in detail in Section 6.2.1, where direct EPFR quenching and downstream ROS interception are treated as distinct and experimentally separable pathways.

A broader perspective on EPFR formation and localization also affects the interpretation of EPR data [2]. EPR measures the total spin concentration in a sample, but it does not by itself distinguish surface-exposed radicals from bulk-embedded radicals [2]. A reduction in EPR signal after CeO_2_ treatment could therefore reflect either direct quenching of accessible radicals or a change in the radical environment that alters relaxation behavior, rather than a true reduction in total radical content [2]. Complementary methods, such as surface-sensitive EPR using probes of different sizes, or depth-profiling techniques, would be needed to determine the fraction of EPFRs that is accessible to CeO_2_. This issue is incorporated into the experimental framework proposed in Section 7.

Finally, the coexistence of metal-mediated and matrix-stabilized EPFRs means that the radical population in ambient PM_2.5_ is likely heterogeneous not only in chemical type (carbon-centered, semiquinone, phenoxyl, and oxygen-centered) but also in spatial location (surface-exposed versus bulk-embedded) [6,9,11,12,21,22,25]. This heterogeneity reinforces the view that a single “EPFR quenching efficiency” is unlikely to be a meaningful material property; instead, quenching efficiency should be reported as a function of radical accessibility, particle matrix composition, and CeO_2_ spatial distribution relative to the captured particles [2,9,12,42].

**Figure 1:**
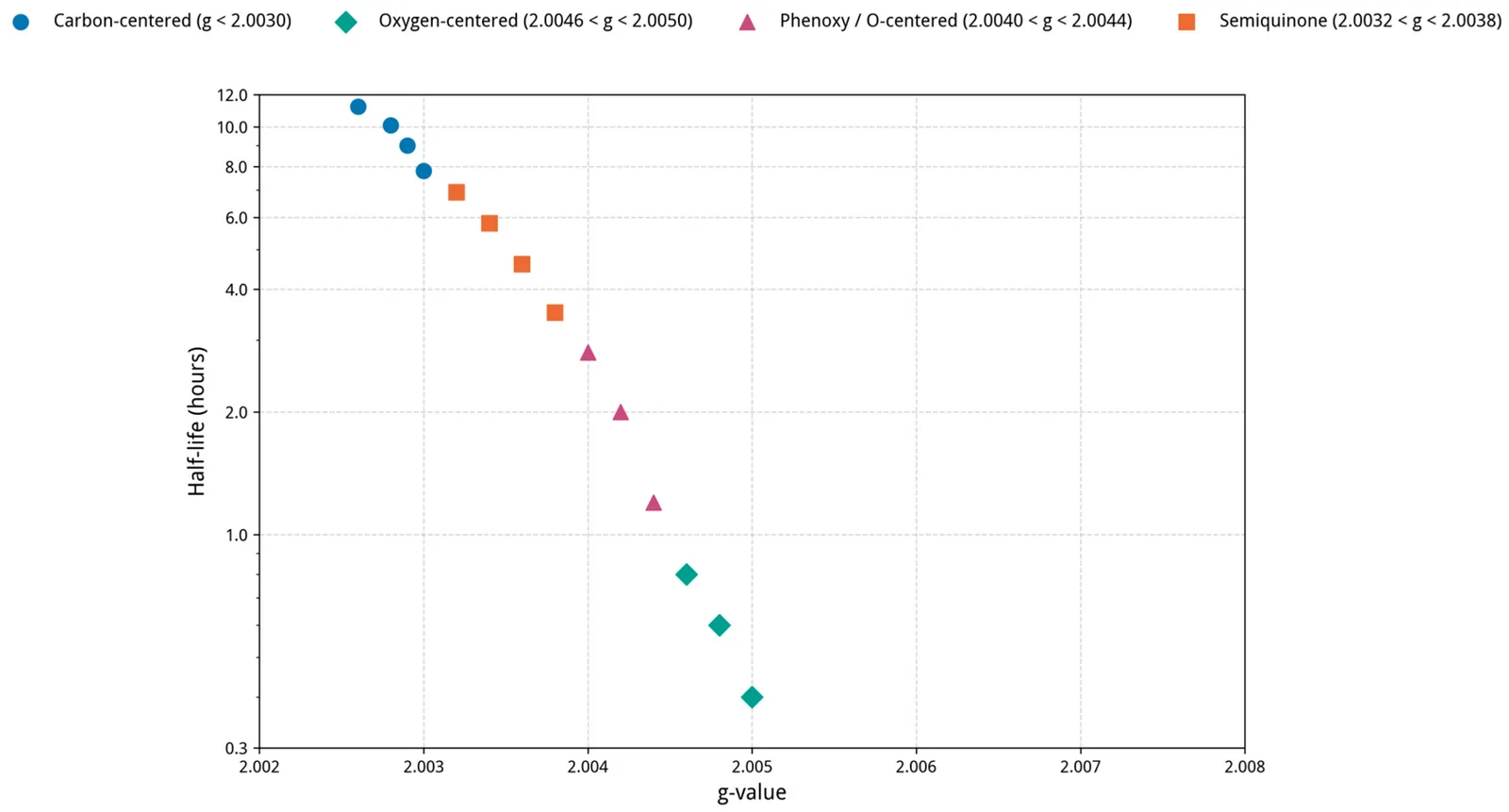
Scatter plot of environmentally persistent free radicals (EPFRs) categorized by g-value, radical type, and short-term half-life. Each data point represents a reported EPFR type detected in ambient particulate matter or combustion-derived particles. The *x*-axis shows the g-value (2.0025–2.0055), which serves as a fingerprint for radical classification: carbon-centered radicals (g < 2.0030; blue circles), semiquinone radicals (2.0030 ≤ g ≤ 2.0040; orange squares), phenoxy and oxygen-centered radicals (2.0035 ≤ g ≤ 2.0045; purple triangles), and oxygen-centered radicals (g > 2.0040; green diamonds). Data are compiled from Wang et al. (2024) [10] and Lu et al. (2025) [25].

The transition-metal pathway involves the adsorption of organic precursors (e.g., phenols, catechols, polycyclic aromatic hydrocarbons) onto metal oxide surfaces, followed by electron transfer from the organic molecule to the metal center (Figure 1). This process generates a radical cation or phenoxyl radical that is stabilized by the oxide lattice. Iron, copper, nickel, and zinc oxides have all been shown to mediate EPFR formation, with half-lives of the resulting radicals ranging from minutes to days depending on the metal and adsorbate. On CuO/silica surfaces, 2-chlorophenoxy-Cu(I), 2-hydroxyphenoxy-Cu(I), and o-semiquinone radical-Cu(I) can form through reactions involving hydroxyl substituents, chlorine substituents, or both. DFT calculations indicate that dissociative adsorption of phenol on Cu(100) and Fe(100) surfaces involves O-H bond cleavage with reaction barriers of approximately 8.2 kcal/mol. Metal speciation plays a decisive role in EPFR stabilization: on CuO, phenol-derived EPFRs exhibit a maximum lifetime of 74 min, whereas on ZnO, two decay modes are observed with 1/e lifetimes of 10 days (fast decay) and 23 days (slow decay) [6].

The organic matrix pathway is particularly relevant for biomass combustion and biochar systems. During pyrolysis, lignin-derived aromatic structures undergo bond cleavage to generate phenoxy radicals (PhO_2_), which can be further stabilized through resonance delocalization within condensed aromatic domains or through formation of covalent adducts with the matrix. Carbon-centered radicals with g-values below 2.0030 are characteristic of this pathway (Figure 1). EPFR concentrations in biochars can range from 10^8^ times lower to 10^9^ times higher than other environmental sources, depending on feedstock, production conditions, and environmental aging. Half-lives of carbon-centered radicals can exceed 100 days in the absence of reactive species, and under certain conditions may reach 100 to 300 days [42].

In ambient PM_2.5_, EPFRs are predominantly oxygen-centered, with g-values typically in the range of 2.0033–2.0048, suggesting contributions from semiquinone and phenoxyl radicals (Figure 1). Combustion sources dominate, accounting for approximately 60% of EPFRs in urban PM_2.5_, with vehicular emissions and solid fuel burning as major contributors. Atmospheric EPFR concentrations over 24 h periods range from 10^19^ to 10^22^ spins/g. Secondary EPFRs can form through atmospheric oxidation and metal catalysis, with photooxidation playing a dominant role. Notably, non-aromatic compounds (e.g., polyethylene, polyamide) can also generate secondary EPFRs under certain environmental conditions, with higher water solubility and stronger ROS-generation efficiency [43].

### 3.2. The Catalytic ROS Generation Cycle

The toxicity of EPFRs is not simply a function of the radical itself but of its capacity to catalyze ROS production. EPFRs undergo redox cycling within aqueous solutions and in biological tissues/fluids, triggering production of ROS comprised primarily of hydroxyl, superoxide, and peroxyl radicals [14].

The proposed mechanism involves a redox cycle. First, surface-bound EPFRs deprotonate and reduce molecular oxygen, generating superoxide:

(1) EPFR + O_2_ → EPFR^+^ + O_2_^−^

Superoxide then undergoes dismutation to H_2_O_2_, either spontaneously or catalyzed by transition metals:

(2) 2 O_2_^−^ + 2H^+^ → H_2_O_2_ + O_2_

Finally, Fenton chemistry between H_2_O_2_ and reduced transition metals (e.g., Fe^2+^, Cu^+^) generates hydroxyl radical:

(3) Fe^2+^ + H_2_O_2_ → Fe^3+^ + ·OH + OH^−^

The EPFR is regenerated through reduction by biological electron donors (e.g., ascorbate, glutathione), allowing the cycle to continue. This catalytic nature explains why EPFRs can produce far more ·OH than would be expected from their initial concentration alone. Experimental measurements and calculations suggest that a single EPFR can generate approximately 10 ·OH radicals, indicating a catalytic rather than stoichiometric process. The ·OH produced is most likely attached to the particle surface rather than acting freely in solution [12].

EPR spin-trapping experiments have directly confirmed this mechanism. Using DMPO as the spin trap, the 4-line EPR spectrum of the DMPO-OH adduct was detected in PBS solutions of both EPFR-bearing particles and control particles. Although the initial difference was modest, the DMPO-OH adduct generated by EPFR particles gradually became twice that of the control as incubation time increased, with maximum difference observed after more than 12 h of incubation. Transition metals play a critical role in modulating EPFR-driven ROS generation. Traffic-source enrichment of Fe and Cu strongly promotes Fenton-driven ·OH generation, whereas marine-source signatures of Mg, V, and Zn favor O_2_^−^ and H_2_O_2_ pathways. This implies that the oxidative potential of EPFRs depends not only on their concentration but also on the species and valence states of coexisting metals [44].

### 3.3. Implications for Filtration Design

The catalytic, sustained nature of EPFR-driven ROS generation has a direct implication for filtration: removing the particle from the airstream does not eliminate its radical activity. A captured particle retains its EPFR content and its capacity to generate ·OH upon contact with biological fluids [45].

The decay behavior of EPFRs in PM_2.5_ further reinforces this conclusion. Freshly collected PM_2.5_ samples showed several decay patterns: 54 samples decayed quickly and then slowly, 27 decayed slowly throughout, 21 showed no decay, and 12 decayed quickly and then stopped. The initial g-factor was 2.0035 ± 0.0004, suggesting PAH-derived carbon-centered radicals or semiquinone-type radicals in complex matrices. Persistent Fe^3+^ signals (g ≈ 2.1) and Mn^2+^ signals were also detected in the samples, and these paramagnetic metals were not degraded during EPFR decay. This finding has important implications for filtration design: even if some EPFRs decay over time, the transition metals coexisting with EPFRs remain active and can continue to catalyze Fenton reactions to generate ·OH [46]. Although Fe is the most widely studied Fenton-active metal in atmospheric particles, Mn(II) can also participate in Fenton-like chemistry [24,44]. Mn(II) reacts with H_2_O_2_ to produce ·OH and Mn(III), and the resulting Mn(III) can be reduced back to Mn(II) by superoxide or organic electron donors, allowing catalytic cycling to continue. The redox potential and reaction kinetics of Mn(II)/Mn(III) differ from those of Fe(II)/Fe(III), but the overall consequence for particle oxidative potential is similar: coexisting redox-active metals can sustain ROS generation even when the original EPFR population has partially decayed [3,16,24,44,45,46]. This reinforces the argument that filtration materials designed to address EPFR-related toxicity should consider not only the radicals themselves but also the metal species that catalyze ROS generation in their vicinity [3,12,16,24,44,45,46].

Furthermore, the filter environment, with its accumulated particle loading and potential for moisture condensation, may create conditions favorable for radical cycling. The stabilization of EPFRs on particle surfaces benefits from the additional stability provided by the oxide lattice, enabling these radicals to persist in the environment. When particles are captured on a filtration membrane, their surface EPFRs remain exposed to oxygen and moisture in the airflow, and the substrates for the catalytic cycle (O_2_, H_2_O) are not removed. A filter that actively quenches EPFRs, or that intercepts the ROS generated by them, would represent a functional advance beyond passive interception. This functional objective is fundamentally different from the passive logic of existing filtration technologies: current technologies optimize how many particles are captured, whereas the new function should answer whether the captured particles remain toxic [12].

## 4. CeO_2_ as a Radical Scavenging Material

### 4.1. Mechanistic Basis: The Ce^3+^/Ce^4+^ Redox Cycle

The radical scavenging activity of CeO_2_ derives from its fluorite crystal structure and the facile interconversion between Ce^3+^ and Ce^4+^. Cerium is the most abundant rare earth element and exists in +3 and +4 oxidation states. In CeO_2_, cerium atoms form a fluorite crystal structure with eight oxygen atoms surrounding each cerium atom. This structure is prone to internal vacancy defects, with a portion of cerium present as Ce^3+^ and positive charges compensated for by oxygen vacancies. The surface of CeO_2_ nanoparticles typically contains a mixture of both oxidation states, and the Ce^3+^/Ce^4+^ ratio is a key determinant of catalytic activity, with distinct optimal ratios for different reactions. The radical scavenging reactions of CeO_2_ can be summarized as follows [27].

For superoxide (SOD-like activity, favored at higher Ce^3+^/Ce^4+^ ratios):

(4) O_2_^−^ + Ce^4+^ → O_2_ + Ce^3+^

For hydrogen peroxide (CAT-like activity, favored at lower Ce^3+^/Ce^4+^ ratios):

(5) H_2_O_2_ + 2Ce^4+^ → O_2_ + 2Ce^3+^ + 2H^+^

(6) H_2_O_2_ + 2Ce^3+^ + 2H^+^ → 2H_2_O + 2Ce^4+^

For hydroxyl radical (direct scavenging):

(7) Ce_2_O_3_ + 2·OH → 2CeO_2_ + H_2_O

The reverse reaction, regeneration of Ce^3+^ from Ce^4+^, occurs through interaction with water or other reducing species:

(8) 2CeO_2_ + H_2_O → Ce_2_O_3_ + ½O_2_ + 2H^+^

This regeneration is the basis for the self-sustaining antioxidant activity of CeO_2_. The catalytic activity of cerium oxide is due to the transition between Ce^4+^ and Ce^3+^ ions, and the lattice structure also exhibits oxygen vacancies, which can convert between CeO_2_ and CeO_2−x_. In experimental validation, CeO_2_ nanoparticles treated with H_2_O_2_ showed a decrease in the Ce^3+^/Ce^4+^ ratio from approximately 0.64 to 0.13, followed by spontaneous recovery over six days to the original ratio, accompanied by a reversal of the visible color change. This demonstrates that the material can cycle between oxidized and reduced states without irreversible degradation [29].

The Ce^3+^/Ce^4+^ ratio is not a fixed material property but can be tuned by synthesis conditions. CeO_2_ nanoparticles with different morphologies and crystal facets expose different proportions of Ce^3+^ sites. The number of Ce^3+^ sites increases as nanoparticle diameter decreases and oxygen atoms are lost (oxygen vacancies), making size control a critical design parameter. This tunability is directly relevant to filtration applications, where the optimal Ce^3+^/Ce^4+^ ratio may differ from that optimized for biomedical applications [47].

### 4.2. Evidence for Radical Scavenging

Direct experimental evidence for CeO_2_-mediated ·OH scavenging comes from EPR spin-trapping studies. Using DMPO as a spin trap, CeO_2_ has been shown to reduce the ·OH signal generated by Fenton chemistry in a concentration-dependent manner. Importantly, this activity is retained after prolonged immersion in phosphate-buffered saline, indicating that the catalytic sites remain accessible and active over extended periods [48].

**Figure 2:**
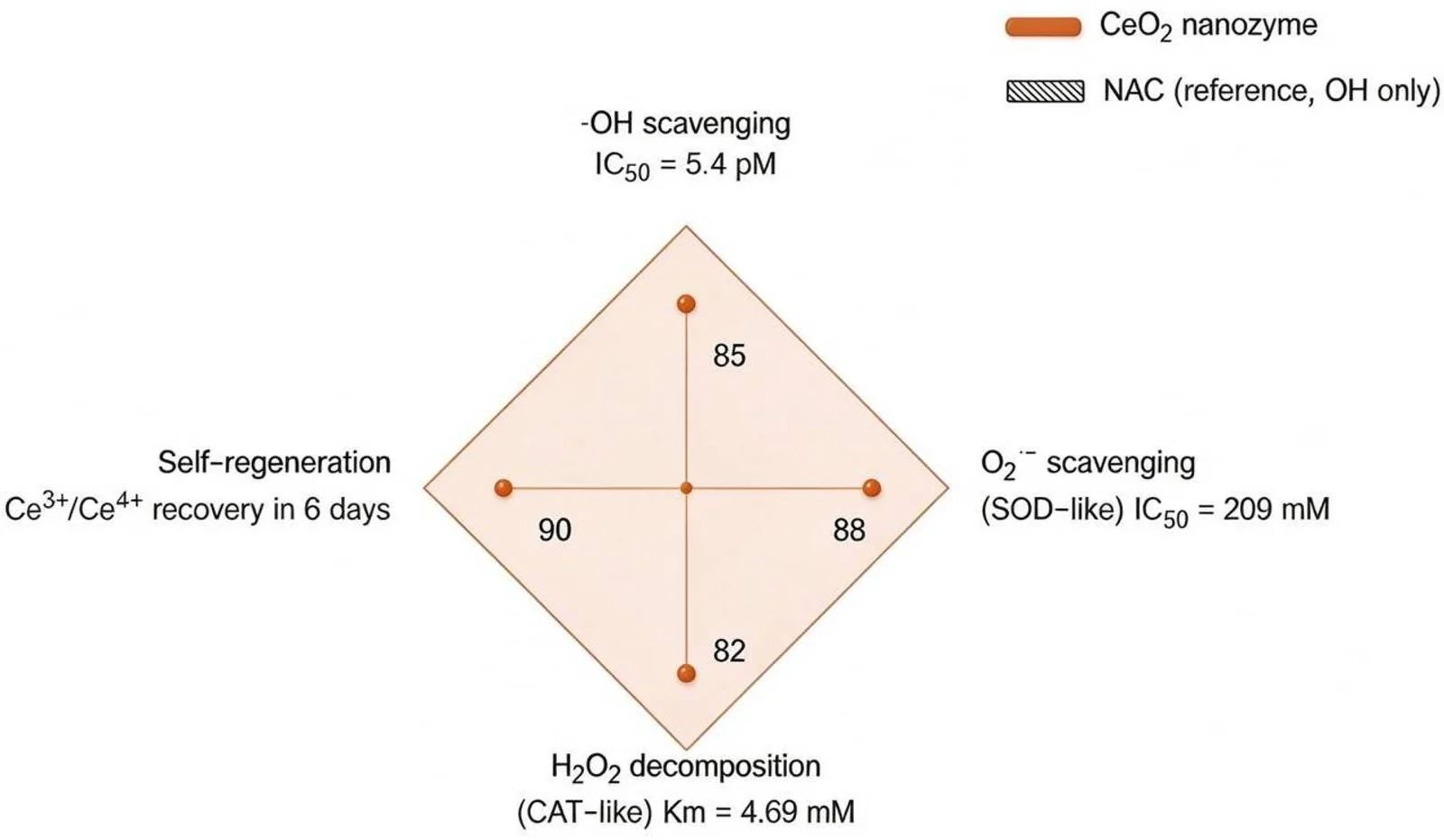
Radar chart of CeO_2_ multi-enzyme reactive oxygen species (ROS) scavenging activity and self-regeneration capacity. Four axes represent distinct catalytic antioxidant functions of CeO_2_ nanozymes: (i) hydroxyl radical (·OH) scavenging, expressed as IC_50_ = 5.4 μM; (ii) superoxide anion (O_2_^−^) scavenging/superoxide dismutase (SOD)-like activity, expressed as IC_50_ = 20.9 nM; (iii) hydrogen peroxide (H_2_O_2_) decomposition/catalase (CAT)-like activity, expressed as Km = 4.69 mM; and (iv) self-regeneration capacity, defined as the time required for spontaneous recovery of the Ce^3+^/Ce^4+^ ratio after oxidative treatment (6 days). All values are normalized to a 0–100 scale, where higher scores indicate stronger scavenging activity or faster regeneration. The blue solid polygon represents CeO_2_ nanozyme performance; the grey dashed polygon represents N-acetylcysteine (NAC) as a reference antioxidant, for which only ·OH scavenging data are available. Normalized values for CeO_2_ are 85 (·OH), 88 (O_2_^−^), 82 (H_2_O_2_), and 90 (self-regeneration). Data are derived from Bai et al. (2024) [26] and Liu et al. (2021) [47].

The SOD-like activity of CeO_2_ has been demonstrated using riboflavin photoreduction to generate superoxide, with CeO_2_ effectively competing with nitroblue tetrazolium for O_2_^−^ (Figure 2). The catalytic mechanism involves Ce^4+^ accepting an electron from O_2_^−^ to form Ce^3+^, with the reaction following first-order kinetics with respect to O_2_^−^ concentration. Notably, the SOD-like activity of CeO_2_ is not blocked by surface coatings: the IC_50_ for SOD-like activity was found to be identical (20.9 nM) for bare CeO_2_ and for polymer-coated CeO_2_, suggesting that superoxide anion can compete with surface coatings for access to Ce^3+^ sites. This has direct implications for filtration applications, where CeO_2_ may be embedded in or coated onto polymer matrices [34].

The CAT-like activity, measured as oxygen evolution from H_2_O_2_ decomposition, shows a similar dependence on the Ce^3+^/Ce^4+^ ratio, with preparations having higher Ce^4+^ content exhibiting greater CAT-like activity. Multi-enzyme activity of CeO_2_ has been quantified: catalytic rate constants for SOD-like activity range from ~3.5 × 10^5^ to 3.5 × 10^6^/(M·s) for nanoceria of different sizes. The catalase-like activity of porous CeO_2_ has a Km of 4.69 mM and a Vmax of 11.7 × 10^−7^ M/s, with a catalytic efficiency (kcat/Km) of 2.60 × 10^8^ M^−1^ s^−1^. Visible light irradiation (405/450 nm) enhances the catalytic efficiency by 105–123%, an effect attributed to photo-induced Ce^3+^/Ce^4+^ cycling. This light-enhancement effect may be relevant to indoor filtration applications, where ambient lighting could contribute to radical scavenging activity [30].

Beyond these enzyme-mimetic activities, CeO_2_ has been shown to directly scavenge nitric oxide radical (·NO), expanding its potential relevance to inflammatory conditions. The mechanism involves Ce^3+^ reacting with ·NO to form Ce^4+^ and NO^−^, followed by further reaction to produce N_2_O. This broad-spectrum activity against multiple ROS and RNS species distinguishes CeO_2_ from conventional antioxidants, which typically target a single radical type [26,47].

### 4.3. CeO_2_ in Gas-Phase Radical Scavenging

The most relevant precedent for the proposed application is the demonstration that CeO_2_ can scavenge gas-phase radicals. In cigarette smoke, which contains high concentrations of both particulate-phase and gas-phase radicals, CeO_2_-based nanozymes incorporated into filter tips reduced ·OH and H_2_O_2_ levels in the filtered smoke. This finding is significant because it demonstrates that CeO_2_ retains radical scavenging activity under non-aqueous, flowing gas conditions. This environment is fundamentally different from the buffered solutions used in most biomedical studies [26].

The cigarette smoke study provides several insights relevant to PM_2.5_ filtration. First, cigarette smoke is an extreme aerosol environment with radical concentrations orders of magnitude higher than ambient air. If CeO_2_ can function under such conditions, its application to lower-concentration indoor air is mechanistically plausible. Second, the study demonstrates that CeO_2_ can be integrated into a flow-through filter configuration, with the nanozyme immobilized on a fibrous substrate. Third, the radical scavenging occurred at ambient temperature, without the need for external energy input. These three features, namely high radical concentration tolerance, flow-through configuration, and ambient operation, are directly transferable to PM_2.5_ filtration [49].

However, several differences between cigarette smoke and ambient PM_2.5_ filtration must be acknowledged. Cigarette smoke exposure is transient (seconds to minutes), whereas indoor air filtration operates continuously (hours to days). The radical concentration in cigarette smoke is much higher, which may drive reaction kinetics favorably. The humidity and temperature of cigarette smoke differ from indoor air. Therefore, while the cigarette smoke study establishes proof-of-concept for gas-phase radical scavenging by CeO_2_, it does not directly validate performance under PM_2.5_ filtration conditions [49]. This gap between proof-of-concept and application validation is a central theme of the present review.

Another relevant gas-phase application is the use of CeO_2_ in catalytic ozone decomposition. MnO_x_-CeO_2_ composites have been shown to decompose ozone at room temperature, with oxygen vacancies serving as active sites. The mechanism involves ozone adsorption on oxygen vacancies, followed by decomposition to O_2_ and surface oxygen species. While the target O_3_ differs from EPFRs, the underlying principle is analogous, namely the catalytic decomposition of a gas-phase oxidant on CeO_2_ surfaces at ambient temperature [50]. This further supports the feasibility of CeO_2_-based gas-phase radical scavenging.

## 5. CeO_2_ in Air Pollution Control

### 5.1. CeO_2_ in Three-Way Catalysis

The largest-scale application of CeO_2_ in air pollution control is as a component of three-way catalytic converters (TWCs) for gasoline engine exhaust. In this context, however, CeO_2_ is not the primary catalytically active phase. The oxidation of CO and hydrocarbons and the reduction of NOx are catalyzed by platinum-group metals (Pt, Pd, Rh). CeO_2_ serves as an oxygen storage material, buffering the oscillations in air-fuel ratio and widening the operating window over which the noble metals function optimally [40].

**Figure 3:**
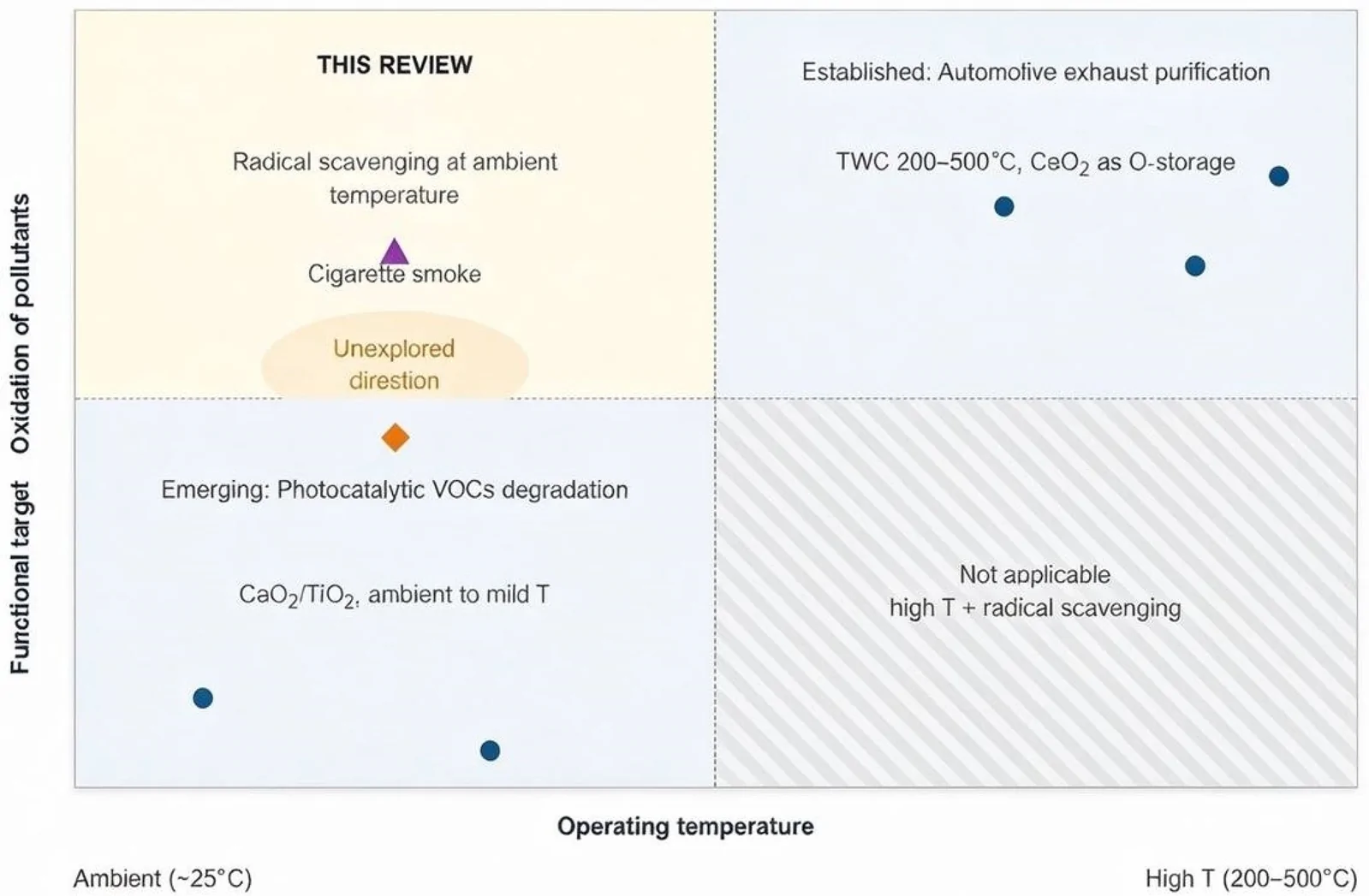
Functional positioning map of CeO_2_ applications in air purification. The two-dimensional quadrant map plots reported CeO_2_ applications along two axes: operating temperature and functional target. The upper-right quadrant (high temperature + oxidation of pollutants) represents the mature, industrially established application of CeO_2_ as an oxygen storage component in three-way catalytic converters (TWC) for automotive exhaust purification, where CeO_2_ operates at 200–500 °C alongside platinum-group metals. The lower-left quadrant (ambient temperature + oxidation of pollutants) represents emerging applications in photocatalytic volatile organic compound (VOC) degradation, including CeO_2_/TiO_2_ heterojunctions and CeO_2_-based electrospun membranes. The upper-left quadrant (ambient temperature + radical scavenging) is the unexplored direction proposed in this review, targeting the quenching of EPFRs and ROS on PM_2.5_ surfaces under filtration conditions. Circles denote established applications; the purple triangle denotes a prior example of gas-phase radical scavenging by CeO_2_ in cigarette smoke; the orange diamond denotes the direction proposed in this review. Data are based on Chen et al. (2025) [49] and Guo et al. (2025) [52].

The functional logic of CeO_2_ in TWCs is therefore fundamentally different from the radical scavenging application proposed here (Figure 3). In TWC, CeO_2_’s redox cycling is harnessed to store and release oxygen at temperatures of 200–500 °C, facilitating the oxidation of pollutants by the noble metal catalysts. Under oxygen-deficient (fuel-rich) conditions, CeO_2_ releases oxygen and is reduced to Ce_2_O_3_, helping to oxidize residual CO and hydrocarbons. Under oxygen-rich (fuel-lean) conditions, Ce_2_O_3_ absorbs oxygen and is restored to CeO_2_, storing oxygen for the next reduction cycle. The elevated temperature is essential: pure CeO_2_ suffers from limited oxygen vacancy formation and short-lived surface-active oxygen species at lower temperatures, necessitating the high-temperature environment of the exhaust stream. At temperatures below approximately 200 °C, the oxygen storage capacity of CeO_2_ drops sharply, and the promotional effect of the material on noble metal catalysis becomes negligible [51].

This distinction matters for the present review because it defines what CeO_2_ is not doing in TWC: it is not the primary catalytic material, it is not operating at ambient temperature, and it is not targeting radicals.

### 5.2. CeO_2_ in Filtration and Adsorption Media

CeO_2_ has been incorporated into various air filtration and purification media, but again with functional objectives distinct from EPFR scavenging. CeO_2_-modified wood fiber filters have been developed for the simultaneous removal of particulate matter and NOx from gasoline engine exhaust. In this application, CeO_2_ serves as a heat-resistant modifier and provides catalytic activity for NOx reduction, with the wood fiber matrix serving as a porous scaffold (Figure 3). The study notes that CeO_2_ can sequester free radicals produced during the thermal decomposition of organic materials, but this occurs in the context of high-temperature exhaust (hundreds of degrees Celsius) and is not the primary design objective [52]. The mention of free radical sequestration is incidental. It appears as a secondary observation within a study focused on thermal stability and NOx removal.

Similarly, CeO_2_/TiO_2_ heterojunctions on activated carbon fiber have been explored for VOC removal, where the CeO_2_ component enhances photocatalytic activity and provides oxygen storage capacity. In this configuration, CeO_2_ facilitates the oxidation of Volatile Organic Compounds (VOCs) through a photocatalytic mechanism, again operating on the principle of oxidizing pollutants rather than quenching existing radicals. Other CeO_2_-based filtration materials include CeO_2_-decorated metal–organic frameworks for enhanced photodynamic therapy (a biomedical application) and CeO_2_/hollow fiber membrane for air filtration, where the function is to enhance particle capture efficiency rather than to detoxify captured particles [53].

A key observation from the literature is that no study has systematically tested whether CeO_2_ can quench EPFRs on PM_2.5_ surfaces. The studies that come closest, such as the wood fiber filter study that mentions radical sequestration, address this phenomenon in the context of high-temperature thermal decomposition rather than ambient filtration. The functional gap is therefore not merely a matter of degree but of kind: existing CeO_2_ filtration applications are designed for either high-temperature oxidation or enhanced physical capture, not for ambient-temperature chemical detoxification of captured radicals [54].

### 5.3. High-Temperature Oxidation vs. Ambient Radical Scavenging

The common thread in existing CeO_2_ air pollution control applications is a reliance on elevated temperatures to drive catalytic oxidation reactions. The function of CeO_2_ is to facilitate the conversion of pollutants (CO, hydrocarbons, NOx, and VOCs) into less harmful products through oxidation [41].

The radical scavenging function proposed in this review operates on a different principle (Figure 3). Rather than oxidizing a pollutant, the objective is to quench an existing radical and thereby terminate the catalytic cycle that generates ROS. This is a reduction reaction, not an oxidation, and it occurs at ambient or near-ambient temperatures. The target is not a stable molecule but a reactive intermediate. This distinction has implications for material design. A CeO_2_ formulation optimized for high-temperature oxidation (e.g., with high oxygen storage capacity and thermal stability) may not be optimal for ambient radical scavenging, where surface Ce^3+^ content and radical accessibility are more critical. The key insight is that the success of CeO_2_ in high-temperature air pollution control does not validate its use for ambient radical scavenging. These are separate functional domains. The former is a mature, industrially established application; the latter is an unexplored hypothesis. Conflating the two would obscure the genuine novelty of the proposed direction and overstate the readiness of CeO_2_ for filtration applications [42].

## 6. The Gap in Using CeO_2_ for EPFR Scavenging in Filtration

### 6.1. The Unaddressed Interface

Despite the mechanistic rationale and relevant precedents, no study has systematically investigated whether CeO_2_ can quench EPFRs on PM_2.5_ surfaces. EPFR research has focused on formation, environmental distribution, and toxicological effects, whereas CeO_2_ radical-scavenging research has focused on biomedical applications. This gap is significant because the two processes share a common redox logic. EPFRs on PM_2.5_ generate ROS through redox cycling, and CeO_2_ scavenges ROS through redox cycling. The unresolved question is whether CeO_2_ can intervene in the EPFR catalytic cycle, either by directly quenching the EPFR through electron transfer or by intercepting the ROS (O_2_^−^, H_2_O_2_, ·OH) as they are generated. If either function can be achieved under filtration conditions, it would extend air filtration from physical interception to active detoxification [1,3,26].

### 6.2. Critical Unknowns in CeO_2_-Based EPFR Scavenging

As discussed in Section 3.1, EPFRs in ambient PM_2.5_ are not necessarily confined to particle surfaces [9,11,12,42]. A fraction of the radical population may be embedded within the organic matrix or within internal pores, particularly in particles formed from incompletely combusted fuels with low metal content [4,9,10,11,12,42,43]. This spatial heterogeneity is directly relevant to the present discussion: direct quenching by CeO_2_ requires that the radical be accessible at the particle–air interface, whereas ROS interception can occur even when the radical itself is inaccessible [9,12,45]. Therefore, the relative importance of direct quenching versus ROS interception is expected to depend not only on the chemical type of the EPFR but also on its spatial location within the particle [9,11,12,42,45]. Addressing this gap requires resolving several issues that remain unaddressed in the literature.

#### 6.2.1. Direct EPFR Quenching Versus Downstream ROS Interception

A central mechanistic question is whether CeO_2_ primarily acts by directly quenching matrix-embedded EPFRs on heterogeneous PM_2.5_ surfaces or by intercepting the ROS that these radicals catalytically generate downstream [9,11,12,26,32,42,45,55,56]. The distinction is not semantic; it determines both the required spatial architecture of the filter and the interpretation of any measured detoxification effect.

Direct EPFR quenching requires that CeO_2_ establish electronic contact with a stabilized radical that may be embedded within an organic matrix, adsorbed at a metal-oxide interface, or sequestered in micropores of the particle [6,7,9,11,12,42]. In ambient PM_2.5_, EPFRs are predominantly oxygen-centered, with g-values of approximately 2.0033–2.0048, consistent with semiquinone and phenoxyl radicals, although carbon-centered radicals also occur in combustion-derived and biomass-burning particles [11,12,25,43,45,46]. These radicals are not freely diffusing species; they are stabilized by resonance delocalization, chelation to transition-metal centers, or incorporation into condensed aromatic domains [6,7,9,12,42,43]. Therefore, direct quenching would require CeO_2_ to overcome steric shielding by the organic matrix, compete with native metal centers for radical access, and satisfy redox-potential matching with different EPFR classes [6,7,9,12,26,27,28,29,30,32,33,34,35,36,42,47,48,56]. For carbon-centered radicals with low g-values, one-electron oxidation by Ce^4+^ may be thermodynamically unfavorable; for oxygen-centered semiquinone radicals, Ce^4+^/Ce^3+^ cycling may be better poised to accept or donate electrons [11,12,25,26,29,30,32,47,48,56]. This heterogeneity means that a single “EPFR quenching” efficiency is unlikely to exist; rather, quenching probability should be treated as a function of radical type, matrix composition, and CeO_2_ surface state [9,11,12,26,27,29,30,33,34,35,36,42,47].

Downstream ROS interception is mechanistically less demanding in terms of physical access [26,32,45,49,56]. If CeO_2_ cannot directly contact matrix-embedded EPFRs, it may still scavenge the superoxide, hydrogen peroxide, and hydroxyl radicals produced by EPFR redox cycling [26,32,33,36,45,47,48,49,56]. In this scenario, CeO_2_ does not need to be in direct contact with the radical-bearing particle; it must instead be positioned where the ROS flux is highest [40,49,50,52]. Because EPFR-driven ROS generation is catalytic and sustained, and because a single EPFR can generate multiple hydroxyl radicals, intercepting even a fraction of the ROS flux could produce a measurable reduction in oxidative potential [3,12,16,24,44,45]. This pathway would favor a layered filter architecture in which a CeO_2_-containing layer is placed downstream of the particle-capture layer, so that the airflow carries ROS toward the scavenger while particles remain immobilized upstream [40,49,50,52].

These two mechanisms are not mutually exclusive [26,32,49]. A CeO_2_-modified filter may quench a fraction of accessible surface EPFRs directly while simultaneously intercepting ROS generated by inaccessible radicals [9,12,26,32,42,49]. The practical question is therefore not “which mechanism is correct?” but “what is the relative contribution of each pathway under realistic filtration conditions?” Resolving this requires experiments that independently vary the spatial proximity between CeO_2_ and EPFR-bearing particles [2,16,24,55]. For example, direct quenching can be tested by mixing CeO_2_ nanoparticles with EPFR-bearing PM_2.5_ under controlled contact conditions and measuring the decay of the EPFR signal by EPR [2,26,32,45,46,49,55]. ROS interception can be tested by separating CeO_2_ from the particle layer with a diffusion barrier that allows gas-phase ROS to pass but prevents particle contact [16,24,49,50]. Comparing the two configurations would allow the direct-quenching and ROS-interception contributions to be disentangled [2,16,24,55].

The answer has direct design consequences. If direct quenching dominates, CeO_2_ must be integrated into the particle-capture layer or coated onto the fibers that retain PM_2.5_, and the material must be engineered to maximize contact with captured particles [26,32,40,52]. If ROS interception dominates, CeO_2_ can be deployed as a separate downstream layer, and the design objective shifts to maximizing ROS residence time and active-site accessibility rather than particle–catalyst contact [40,49,50,52]. If both pathways contribute, a hierarchical filter with CeO_2_ in both the capture and downstream layers may be optimal [40,49,52]. At present, none of these possibilities has been experimentally tested for EPFR-bearing PM_2.5_, which is precisely why the hypothesis remains open [5,54].

#### 6.2.2. Humidity Effects

Relative humidity is a critical but poorly constrained variable for CeO_2_-based EPFR scavenging [26,29,30,47,48,57]. In biomedical studies, CeO_2_ radical scavenging is typically evaluated in aqueous buffers, where water is abundant, and proton transfer is not limiting [26,32,33,36,47,48,57]. In air filtration, however, humidity ranges from very dry indoor air in winter to near-saturation conditions in humid climates, and the CeO_2_ surface may be covered by a submonolayer, monolayer, or multilayer of adsorbed water depending on RH [40].

Three opposing effects must be considered. First, water molecules can compete with O_2_, superoxide, H_2_O_2_, and hydroxyl radicals for adsorption on Ce^3+^ and oxygen-vacancy sites [26,29,30,47,48,57]. At high RH, a continuous water film may form on the CeO_2_ surface, blocking access to active sites and reducing the probability that a gas-phase radical or ROS intermediate reaches a catalytically active Ce^3+^ center [29,30,47,57].

Second, water is a reactant in the CeO_2_ self-regeneration reaction. The re-reduction of Ce^4+^ to Ce^3+^ can proceed through interaction with water, as shown in reaction (8), and proton transfer is also involved in catalase-like and SOD-like cycling [26,29,30,32,47,48,57]. At very low RH, the absence of adsorbed water may limit the regeneration of Ce^3+^ and cause the material to accumulate in the oxidized Ce^4+^ state, reducing its long-term radical-scavenging capacity [29,47,48,57]. Thus, some humidity is likely necessary for sustained cycling [29,47,48,57].

Third, water can alter the mobility and speciation of ROS. Hydroxyl radicals are extremely short-lived and react near their site of generation, whereas superoxide and H_2_O_2_ can diffuse further in the presence of a water film [17,45]. At intermediate RH, a thin adsorbed water layer may facilitate proton-coupled electron transfer and allow ROS to reach CeO_2_ active sites, whereas at very high RH the same water layer may become a diffusion barrier for gas-phase radicals and promote Ce ion leaching [29,47,57].

The net effect is therefore non-monotonic: scavenging efficiency may be low at very low RH because self-regeneration is slow, increase at intermediate RH because proton transfer and ROS mobility are enhanced, and decrease again at high RH because active sites are blocked and Ce leaching accelerates [29,47,57]. The optimal humidity window for CeO_2_-based EPFR scavenging is currently unknown. It cannot be inferred from aqueous biomedical studies, because those systems are effectively at 100% RH and do not capture the competing adsorption and diffusion effects that dominate in air filtration [26,32,47]. Systematic experiments at controlled RH—for example, 20%, 40%, 60%, and 80%—are needed to map the humidity response and to determine whether the optimal window overlaps with realistic indoor conditions [2,16,24,55].

#### 6.2.3. Temperature Effects

Most CeO_2_ nanozyme studies are performed at 37 °C, reflecting physiological conditions [26,32,33,36,47,48]. Indoor air filtration, by contrast, occurs at 20–30 °C, and in some cases lower [40]. The temperature difference of 7–17 °C is modest in absolute terms but can significantly affect reaction kinetics because radical scavenging and catalytic cycling are activated processes. If the activation energy for CeO_2_-mediated superoxide dismutation, H_2_O_2_ decomposition, or hydroxyl radical scavenging is on the order of 20–50 kJ/mol, a decrease from 37 °C to 25 °C could reduce the rate constant by approximately 20–40%. If the activation energy is higher, the reduction could be more pronounced.

At present, the activation energies for CeO_2_-mediated radical scavenging under gas-phase or near-ambient filtration conditions have not been measured [47,48]. This is a critical gap because a material that performs well in a 37 °C buffered suspension may perform substantially worse at 25 °C in air [26,32,47,48]. The temperature effect also interacts with humidity: at lower temperatures, the saturation vapor pressure of water decreases, so a given absolute humidity corresponds to a higher RH, which may exacerbate site blocking [29,57]. Conversely, lower temperature may increase the adsorption equilibrium constant for ROS on CeO_2_, partially compensating for slower intrinsic kinetics [29,57].

The practical implication is that CeO_2_ formulations optimized at physiological temperature may not be optimal for indoor filtration [26,32,47,48]. Experimental testing should therefore include temperature as an explicit variable, ideally spanning 15–35 °C, and should report Arrhenius parameters or at least apparent rate constants at relevant temperatures [2,16,24,47,55]. Without such data, claims about ambient-temperature performance remain extrapolations from biomedical conditions [26,32,47,48].

#### 6.2.4. Airflow, Contact Time, and the Turnover-Rate Constraint

Airflow determines the contact time between radicals and CeO_2_ active sites. In a typical indoor air filter, the face velocity is approximately 0.1–0.5 m/s, and the residence time of air within the filter medium may be only milliseconds to seconds [40]. This is orders of magnitude shorter than the incubation times used in most CeO_2_ radical-scavenging studies, which range from minutes to hours [26,32,47,48].

The key question is whether CeO_2_ catalytic turnover is fast enough to produce meaningful radical quenching within the available contact time. The catalytic rate constants reported for CeO_2_ nanozymes provide a useful starting point. SOD-like rate constants range from approximately 3.5 × 10^5^ to 3.5 × 10^6^/(M·s) [47,48], and the catalytic efficiency for catalase-like activity has been reported as approximately 2.60 × 10^8^/(M·s) [30]. These values are high in absolute terms, but they were measured in aqueous solution with well-mixed reactants [30,47,48]. In a flow-through filter, the effective rate depends on the mass-transfer coefficient for ROS transport from the gas phase to the CeO_2_ surface, the surface concentration of accessible Ce^3+^ sites, and the intrinsic turnover frequency [29,30,47,48].

A simple order-of-magnitude analysis illustrates the challenge. If the CeO_2_ loading is 1 mg/cm^2^ and the specific surface area is 100 m^2^/g, the total surface area per unit filter area is approximately 10^4^ cm^2^. If only 1% of this area is catalytically active and the turnover frequency is 1/s per active site, the surface can process on the order of 10^18^ radical events per second per square meter, which is far greater than the ROS flux expected from typical indoor PM_2.5_ EPFR concentrations [45]. However, this calculation assumes that every ROS molecule reaches an active site, which is unlikely at millisecond contact times. The actual scavenging efficiency will be limited by mass transfer and by the fraction of active sites that remain accessible under humidity and dust loading [29,40,47].

Therefore, contact time is not merely an engineering parameter; it is a mechanistic constraint that determines whether the proposed function is kinetically feasible. Experimental validation should measure radical scavenging as a function of face velocity and filter thickness, and should report the dimensionless contact time or Damköhler number, so that performance can be compared across different filter configurations [2,16,24,40,55].

The short contact time in indoor filtration is arguably the most severe kinetic challenge for the proposed hypothesis. Even if CeO_2_ is thermodynamically capable of quenching EPFR-derived ROS, the reaction must occur within the residence time of air in the filter. For a filter with a face velocity of 0.3 m/s and a thickness of 1 mm, the nominal residence time is approximately 3 ms. If the filter is pleated and the airflow path is longer, the residence time may increase to tens of milliseconds, but it remains far shorter than the minutes-to-hours timescales of typical CeO_2_ nanozyme assays [26,32,40,47,48].

Three factors could mitigate this challenge. First, the intrinsic reactivity of hydroxyl radicals is extremely high; if CeO_2_ is present at the point of ROS generation, the reaction may be diffusion-limited rather than activation-limited [17,45]. Second, the catalytic nature of CeO_2_ means that each active site can turnover many times, so a relatively small number of accessible sites may suffice to process a significant ROS flux [26,32,47,48]. Third, the EPFR-driven ROS generation is itself continuous, so the filter does not need to quench a single pulse of radicals; it must instead maintain a steady-state scavenging rate that matches the ROS generation rate [45].

Nevertheless, the kinetic feasibility of the proposed function cannot be assumed. It must be tested experimentally by measuring radical scavenging at realistic face velocities and by comparing the observed rate with the ROS generation rate of captured PM_2.5_ [2,16,24,45,55]. If the scavenging rate is limited by mass transfer, then increasing CeO_2_ loading or surface area will not help; the filter design must instead maximize contact between the airflow and the CeO_2_ surface [40]. If the scavenging rate is limited by intrinsic turnover, then material optimization should focus on increasing the density of accessible Ce^3+^ sites and reducing activation barriers [29,30,47,48]. Distinguishing between these regimes is essential for rational filter design [40,47].

#### 6.2.5. Long-Term Stability and Deactivation Mechanisms Under Realistic Filtration Conditions

The self-regenerating Ce^3+^/Ce^4+^ cycle implies that CeO_2_ is not stoichiometrically consumed, but this does not mean that it remains indefinitely active [26,29,30,32,47,48,57]. Under realistic filtration conditions, several deactivation pathways can degrade performance over time, and these must be evaluated before CeO_2_-based EPFR scavenging can be considered a viable filter function [40,52].

As PM_2.5_ accumulates on the filter, it forms a dust cake that can cover the CeO_2_ surface and block access to active sites [40,52]. The effect is not simply a reduction in geometric surface area; the deposited particles may also alter the local humidity and pH environment, create diffusion barriers for ROS, and physically separate CeO_2_ from the radicals it is intended to quench [40]. If CeO_2_ is embedded within the filter fibers, dust loading may be less detrimental because the fibers remain partially exposed; if CeO_2_ is coated on the upstream surface, dust masking may be severe [40,52]. The rate of activity loss should be measured as a function of particle loading, and the filter should be designed so that CeO_2_ remains accessible even after substantial dust accumulation [40,52].

Indoor and outdoor air contains SO_2_, NOx, VOCs, and other reactive gases that can adsorb strongly on CeO_2_ surfaces and poison active sites [37,38,39,50,51]. SO_2_ is particularly problematic because it can form stable sulfates on CeO_2_, blocking oxygen vacancies and reducing the Ce^3+^ fraction [37,38,39,51]. NOx can compete with O_2_ and ROS for adsorption sites [37,38,50], and VOCs can form carbonaceous deposits that mask active sites [39]. The severity of poisoning depends on the concentrations of these co-pollutants, the exposure duration, and the affinity of CeO_2_ for each species [37,38,39,50,51]. In indoor environments, SO_2_ and NOx concentrations are typically lower than in combustion exhaust, but chronic exposure over weeks to months may still cause cumulative deactivation [40]. Accelerated aging experiments with controlled co-pollutant exposure are needed to quantify these effects [2,16,24,37,55].

If the re-reduction of Ce^4+^ to Ce^3+^ is slow relative to the oxidation rate, the material may accumulate in the oxidized state and lose radical-scavenging activity [29,47,48,57]. This is most likely under dry conditions, where water is unavailable for the regeneration reaction, or under high ROS flux, where the oxidation rate exceeds the regeneration rate [29,45,57]. The self-regeneration time reported in biomedical studies—approximately six days for recovery of the Ce^3+^/Ce^4+^ ratio after H_2_O_2_ treatment—is long compared with the contact time in a filter, but it is short compared with the service life of a filter [29]. The relevant question is whether the material reaches a steady-state Ce^3+^/Ce^4+^ ratio under continuous radical exposure, and whether that steady state retains sufficient activity [29,47,57].

Leaching of cerium ions under humid conditions. CeO_2_ is generally considered insoluble, but at high humidity and in the presence of acidic or complexing species, trace amounts of Ce^3+^ or Ce^4+^ may leach from the surface [29,57]. Leaching would not only deplete the active material but also introduce cerium into the treated air, raising potential health concerns [29,57]. The leaching rate should be measured under realistic humidity and airflow conditions, and the material should be designed to minimize release of free Ce ions [2,29,40,55,57].

Filters may be subjected to vibration, back-pulsing, and temperature fluctuations during operation and maintenance. CeO_2_ coatings may delaminate, crack, or detach from the substrate [40,52]. The mechanical stability of the CeO_2_–substrate interface should therefore be tested under conditions that mimic real filter handling and operation [40,52]. Taken together, these deactivation pathways define the long-term performance envelope of CeO_2_-based EPFR scavenging. A material that performs well in short-term laboratory tests may fail within days or weeks under realistic dust loading and co-pollutant exposure [40,52]. Long-term stability data are therefore not optional; they are essential for assessing whether the proposed function is practically viable [5,40,52,54].

If CeO_2_ cannot efficiently access EPFRs within the particle matrix, it may still intercept the ROS they generate [9,12,26,32,42,45,49,56]. The relative contribution of these two pathways would determine the required CeO_2_ loading and its spatial distribution relative to captured PM. If ROS interception dominates, CeO_2_ must be positioned downstream of the captured particles to intercept the ROS flux [40,49,50,52]. If direct EPFR quenching dominates, CeO_2_ must be in physical contact with the particles [6,9,12,26,32,42]. These scenarios have different implications for filter design: the former could tolerate a layered architecture with CeO_2_ in a separate layer, while the latter requires direct mixing of CeO_2_ with the filtration medium [40,49,50,52]. Distinguishing between these pathways requires experiments that independently vary the spatial proximity between CeO_2_ and EPFR-bearing particles [2,16,24,55].

Airflow, humidity, and temperature differ substantially from the buffered solutions used in most CeO_2_ radical-scavenging studies, and these differences may critically affect performance [26,29,32,33,36,40,47,48,57]. These effects are discussed in detail in Section 6.2.2, Section 6.2.3 and Section 6.2.4, which address humidity, temperature, and contact time, respectively. The self-regenerating Ce^3+^/Ce^4+^ cycle implies that CeO_2_ is not consumed, but this has not been validated under filtration conditions [5,26,29,30,32,47,48,54,57]. The potential deactivation mechanisms are analyzed in Section 6.2.5, which considers irreversible oxidation, poisoning, dust masking, and Ce leaching [29,37,38,39,40,47,50,51,52,57].

## 7. Experimental Framework for Testing the Hypothesis

The central hypothesis of this review, namely that CeO_2_-based materials can chemically quench EPFRs on captured PM_2.5_ under filtration conditions, remains experimentally unvalidated. The following experimental framework is proposed to test it in a staged, falsifiable manner.

### 7.1. Materials and Sample Preparation

EPFR-bearing PM_2.5_ should be collected from representative combustion sources (e.g., biomass burning, vehicle exhaust, and coal combustion) and from ambient urban air [11,12,21,22,43,46]. Source-specific samples are preferable for mechanistic studies because they allow the EPFR type and concentration to be controlled [3,11,12,43]. The EPFR concentration should be quantified by EPR, and the g-value should be recorded to classify the dominant radical type (carbon-centered, semiquinone, phenoxyl, or oxygen-centered) [2,11,12,25,55]. CeO_2_ nanoparticles with well-characterized size, morphology, surface area, and Ce^3+^/Ce^4+^ ratio should be used [26,27,29,30,33,34,35,36,47,48]. The Ce^3+^/Ce^4+^ ratio should be measured by XPS before and after reaction, and oxygen vacancy concentration should be quantified by Raman spectroscopy or EPR [29,30,47,55].

### 7.2. Direct EPFR Quenching Assay

The direct quenching assay should mix EPFR-bearing PM_2.5_ with CeO_2_ nanoparticles in a controlled environment [26,32,45,46,49]. The mixture should be incubated under defined temperature, humidity, and contact time, and the EPFR signal should be measured by EPR at regular intervals [2,55]. A CeO_2_-free control should be run in parallel to account for spontaneous EPFR decay [46]. The key output is the difference in EPR signal intensity between the CeO_2_-treated and control samples as a function of time. A statistically significant, CeO_2_-dependent decrease in signal intensity would provide evidence for direct quenching [2,55].

Because EPFRs in PM_2.5_ are heterogeneous, the EPR spectrum should be deconvoluted to distinguish carbon-centered, semiquinone, and oxygen-centered contributions [11,12,25]. If direct quenching is selective, the intensity of some components may decrease while others remain unchanged. The g-value should be monitored for shifts that indicate changes in the radical environment [2,11,12,25,55]. Spin trapping with 5,5-Dimethyl-1-pyrroline N-oxide (DMPO) or 5-tert-Butoxycarbonyl-5-methyl-1-pyrroline N-oxide (BMPO) should be used to detect short-lived radicals generated during the reaction, and the formation of Ce^3+^ or Ce^4+^ should be monitored by X-ray photoelectron spectroscopy or ultraviolet–visible spectroscopy [2,29,45,55].

### 7.3. ROS Interception Assay

To test whether CeO_2_ intercepts ROS downstream of EPFR-bearing particles, a two-layer configuration should be used. The upstream layer contains EPFR-bearing PM_2.5_ immobilized on a filter substrate; the downstream layer contains CeO_2_ [40,45,49,50,52]. A diffusion barrier that allows gas-phase ROS to pass but prevents particle contact should be placed between the layers [49,50]. The ROS flux reaching the downstream layer should be measured by spin trapping followed by EPR, or by a chemical probe such as terephthalic acid for hydroxyl radicals or Amplex Red for H_2_O_2_ [2,16,24,45,55]. The difference in ROS signal between the CeO_2_-containing and CeO_2_-free downstream layers would quantify the interception efficiency [49,50].

### 7.4. Acellular Oxidative Potential Assays

EPR measurements provide direct evidence of radical quenching, but they do not capture the biological relevance of the treated particles [2,3,16,24,55]. Acellular oxidative potential assays should therefore be used to measure the ability of EPFR-bearing PM_2.5_ to generate ROS after exposure to CeO_2_ [3,16,24,44]. The dithiothreitol (DTT) assay is widely used for PM oxidative potential and measures the rate of DTT consumption, which correlates with the generation of ROS [3,16,24]. The ascorbic acid (AA) assay provides complementary information [16,24]. Both assays should be performed on PM_2.5_ samples before and after CeO_2_ treatment, and the results should be compared with EPR-derived EPFR decay [2,3,16,24,55]. A reduction in DTT or AA consumption would indicate that CeO_2_ treatment reduces the oxidative potential of the particles, even if some EPFRs remain [3,16,24].

### 7.5. Chemiluminescence and Bioluminescence Assays

In addition to EPR and acellular oxidative-potential assays, chemiluminescence and bioluminescence methods should be included as complementary functional assays [2,4,14,15]. Chemiluminescence can be used to monitor ROS generation in real time and to assess whether CeO_2_ treatment reduces the radical flux from EPFR-bearing PM_2.5_ [2,14,15]. Bioluminescence assays using luminescent bacteria can provide an integrative toxicity endpoint that reflects the net oxidative burden of the treated particles [4]. These assays are particularly useful for comparing CeO_2_-treated and untreated samples under realistic humidity, temperature, and airflow conditions, because they respond to the overall ROS-generating capacity rather than to a single radical species [2,4]. Combining EPR, DTT/AA, chemiluminescence, and bioluminescence endpoints would allow the effect of CeO_2_ to be evaluated across molecular, chemical, and biological levels of observation [2,3,4,16,24,55].

### 7.6. Filtration-Environment Testing

The assays described above should be performed under conditions that mimic realistic filtration. Key variables include relative humidity (20–80%), temperature (15–35 °C), face velocity (0.1–0.5 m/s), and contact time (milliseconds to seconds) [26,32,40,47]. A flow-through filter test rig should be used, with CeO_2_-loaded filter media and EPFR-bearing PM_2.5_ introduced upstream [40,49,52]. The ROS concentration downstream should be measured by spin trapping or chemical probes, and the EPFR content of the captured particles should be measured by EPR after exposure [2,45,49,55]. Long-term tests should be conducted over days to weeks with continuous airflow and periodic dust loading, and the Ce^3+^/Ce^4+^ ratio, surface composition, and scavenging efficiency should be monitored over time [29,40,47,52].

### 7.7. Data Interpretation and Falsification Criteria

The hypothesis would be supported if CeO_2_ treatment produces a statistically significant reduction in EPFR signal intensity, ROS generation, or acellular oxidative potential relative to controls, and if this reduction persists under realistic humidity, temperature, and airflow conditions [2,3,16,24,55]. The hypothesis would be falsified if no significant difference is observed, if the effect disappears under realistic conditions, or if the material deactivates rapidly [29,40,47]. Even a negative result would be informative, because it would define the boundary conditions under which CeO_2_-based EPFR scavenging is not feasible.

### 7.8. Reporting Standards

To facilitate comparison across studies, reports should include the following: PM_2.5_ source and composition; EPFR concentration and g-value; CeO_2_ particle size, surface area, and Ce^3+^/Ce^4+^ ratio; experimental temperature, RH, and contact time; CeO_2_ loading and filter architecture; EPR acquisition parameters; and the results of both EPR and acellular assays [2,3,11,12,16,24,25,26,29,40,47,55]. Where possible, rate constants or turnover frequencies should be reported, along with the estimated ROS generation rate of the PM_2.5_ sample [30,45,47,48]. This would allow the scavenging efficiency to be evaluated in kinetic terms rather than as a single percentage reduction [30,47,48].

## 8. Conclusions and Outlook

This review argues that air filtration should be extended from physical interception to active detoxification, and that CeO_2_-based materials provide a mechanistically grounded starting point for this extension. Three observations support this position. EPFRs on PM_2.5_ constitute a health-relevant pollutant class that current filtration technology does not address. CeO_2_ possesses a self-regenerating, broad-spectrum radical-scavenging mechanism validated in both biological and gas-phase contexts. The intersection of these two fields, with CeO_2_ for EPFR scavenging in filtration, remains unexplored.

The next step is experimental. The most direct test would involve loading CeO_2_ onto a filter substrate, capturing EPFR-bearing PM_2.5_, and measuring both the decay of the EPFR signal by EPR and the reduction in ROS-generating capacity by acellular or cellular assays, in the presence and absence of CeO_2_. Such studies would establish whether the mechanistic rationale translates into a measurable functional benefit, and whether the material remains stable under realistic airflow, humidity, and temperature conditions.

Several critical questions remain unresolved and should define the immediate research agenda. First, is the dominant mechanism direct EPFR quenching or downstream ROS interception? This determines whether CeO_2_ must be in physical contact with captured PM_2.5_ or can function as a separate reactive layer. Second, what is the optimal humidity window, given that water can both block active sites and participate in Ce^3+^ regeneration? Third, are CeO_2_ turnover rates fast enough to keep pace with millisecond-to-second contact times in indoor filters? Fourth, how stable is CeO_2_ under long-term dust loading and exposure to SO_2_, NOx, and VOCs? Fifth, can the proposed function be demonstrated using EPR and acellular oxidative-potential assays under realistic filtration conditions? Addressing these questions would move the field from a plausible hypothesis to an experimentally validated filtration function. In addition, future studies should recognize that EPFRs in ambient PM_2.5_ are heterogeneous not only in chemical type but also in spatial location, and that the accessibility of radicals to CeO_2_ is a key determinant of whether direct quenching or downstream ROS interception dominates.

## Data Availability

No new data were created or analyzed in this study. Data sharing is not applicable to this article.
